# Abscisic acid mimic-fluorine derivative 4 alleviates water deficit stress by regulating ABA-responsive genes, proline accumulation, CO2 assimilation, water use efficiency and better nutrient uptake in tomato plants

**DOI:** 10.3389/fpls.2023.1191967

**Published:** 2023-06-08

**Authors:** David Jiménez-Arias, Sarai Morales-Sierra, Emma Suárez, Jorge Lozano-Juste, Alberto Coego, Juan C. Estevez, Andrés A. Borges, Pedro L. Rodriguez

**Affiliations:** ^1^ ISOPlexis, Center for Sustainable Agriculture and Food Technology, Madeira University, Madeira, Portugal; ^2^ Chemical Plant Defence Activators Group, Department of Life Science & Earth, Instituto de Productos Naturales y Agrobiología-CSIC, Avda Astrofísico Francisco Sánchez 3, Canary Islands, Spain; ^3^ Grupo de Biología Vegetal Aplicada, Departamento de Botánica, Ecología y Fisiología Vegetal, Universidad de La Laguna, Avda, Astrofisico Francisco Sánchez, Canary Islands, Spain; ^4^ Instituto de Biología Molecular y Celular de Plantas, Consejo Superior de Investigaciones Científicas, Universidad Politécnica de Valencia, Valencia, Spain; ^5^ Centro Singular de Investigación en Química e Bioloxía Molecular (CiQUS), Departamento de Química Orgánica, Universidade de Santiago de Compostela, Santiago de Compostela, Spain

**Keywords:** abscisic acid, abiotic stress, ABA receptor, agonist, proline, transpiration, water use efficiency, tomato

## Abstract

Water deficit represents a serious limitation for agriculture and both genetic and chemical approaches are being used to cope with this stress and maintain plant yield. Next-generation agrochemicals that control stomatal aperture are promising for controlling water use efficiency. For example, chemical control of abscisic acid (ABA) signaling through ABA-receptor agonists is a powerful method to activate plant adaptation to water deficit. Such agonists are molecules able to bind and activate ABA receptors and, although their development has experienced significant advances in the last decade, few translational studies have been performed in crops. Here, we describe protection by the ABA mimic-fluorine derivative 4 (AMF4) agonist of the vegetative growth in tomato plants subjected to water restriction. Photosynthesis in mock-treated plants is markedly impaired under water deficit conditions, whereas AMF4 treatment notably improves CO_2_ assimilation, the relative plant water content and growth. As expected for an antitranspirant molecule, AMF4 treatment diminishes stomatal conductance and transpiration in the first phase of the experiment; however, when photosynthesis declines in mock-treated plants as stress persists, higher photosynthetic and transpiration parameters are recorded in agonist-treated plants. Additionally, AMF4 increases proline levels over those achieved in mock-treated plants in response to water deficit. Thus water deficit and AMF4 cooperate to upregulate ***P5CS1*** through both ABA-independent and ABA-dependent pathways, and therefore, higher proline levels are produced Finally, analysis of macronutrients reveals higher levels of Ca, K and Mg in AMF4- compared to mock-treated plants subjected to water deficit. Overall, these physiological analyses reveal a protective effect of AMF4 over photosynthesis under water deficit and enhanced water use efficiency after agonist treatment. In summary, AMF4 treatment is a promising approach for farmers to protect the vegetative growth of tomatoes under water deficit stress.

## Introduction

Climate change is probably the greatest challenge that agricultural science research has to face in the coming years. Climate prediction models indicate that agricultural productivity will be significantly affected in the future (Neupane et al., 2022). The expected increase in global average temperature exacerbates the depletion of water resources as climate variability increases and poses a serious threat to reaching global food security (Neupane et al., 2022). Such a goal requires an increase in food production; however, productivity has increased less than expected over the past decade, exacerbated by a dramatic decline in fertile arable land and water availability for agriculture ((Tester and Langridge, 2010; Wang, 2022). Considering that agricultural irrigation accounts for 85% of the world’s water use, proper water management seems to be a crucial point to maintain crop yield (Alcon et al., 2022). Different approaches are being used to increase the efficiency of watering in crops, such as deficit irrigation (Li et al., 2022), breeding (Pandey et al., 2022), genetically modified organisms (Vega Rodríguez et al., 2022) or biostimulants (Bs)-based strategies (Jiménez-Arias et al., 2019; Jiménez-Arias et al., 2022a). Bs are one of the most promising strategies to cope with yield losses due to water deficit stress (Jiménez-Arias et al., 2021). Given that Bs are in some cases complex mixtures (botanical extracts, protein hydrolysates), the study of pure active ingredients is required to understand how Bs confer drought tolerance (García-García et al., 2020).

Enhanced water use efficiency (WUE) is a trait used in plant breeding to select plants that are more tolerant to water deficit (Condon, 2004). ABA modulates plant response against drought stress and exogenous treatment helps to mitigate the deleterious effect of water deficit and increases WUE (Ahmad Lone et al., 2022; Aslam et al., 2022; Hu et al., 2022; Yang et al., 2019). Specifically, some reports have shown that the modulation of ABA responses constitutes a great opportunity to improve the WUE of crop plants (Pizzio et al., 2013; Yang et al., 2019; Hewage et al., 2020; Vaidya and Cutler, 2022). For example, the overexpression (OE) of the ABA-receptor PYL4^A194T^ in barley (*Hordeum vulgare*) enhances drought tolerance (Rodriguez, 2014), or the OE of *Triticum aestivum TaPYL4* results in improved grain production and increased WUE in wheat plants under drought conditions compared to wild type (Mega et al., 2019). In recent work, OE of the *TaPYL1-1B* gene leads to an increase in WUE and drought tolerance in wheat (Mao et al., 2022). Therefore, activation of ABA signaling is a promising approach to increase WUE and yield under drought stress. Constitutive enhancement of ABA signaling by genetic means might be deleterious at certain stages of plant development. Instead, conditional activation of the pathway only when stress occurs might represent a clear advantage (Vaidya et al., 2019; Lozano-Juste et al., 2023). Positive ABA-mediated responses to coping with drought stress have been widely reported, such as stomata closure, waxes thickening, antioxidant system activation, compatible osmolyte accumulation, synthesis of protective proteins, and changes in root architecture and hydrotropism (Dietrich et al., 2017; Hura, 2020; Miao et al., 2021). Taken together, these adaptations enhance yield under drought stress; therefore, conditional (“on demand”) activation of ABA signaling by application of ABA-receptor agonists or those Bs able to activate ABA biosynthesis or signaling might be an effective approach (Vaidya et al., 2019; Jiménez-Arias et al., 2021). Molecules that are able to efficiently dock and activate ABA receptors act as agonists and lead to the activation of the ABA pathway. On the other hand, those that act as antagonists lead to the blockade of ABA receptors and downregulation of the ABA pathway. When water sources are not limiting, ABA-receptor antagonists might enhance crop yield or serve to stimulate seed germination (Miao et al., 2018; Vaidya et al., 2021).

The development of ABA-receptor agonists with improved properties is necessary because the positive effect of exogenous ABA application is rapidly reduced due to the fragility of its structure, which is sensitive to UV light (Gao et al., 2016; Cao et al., 2017). Thus, ABA exposed to UV radiation loses its bioactivity by isomerizing to trans-ABA, which is less active than normal cis-ABA (Gao et al., 2016). Therefore, the half-life of ABA is only 24 min, which results in an increased cost for the field application of ABA (Cao et al., 2017). Moreover, endogenous ABA catabolism is very active because three oxidizing pathways and conjugation with glucose lead to the inactivation of ABA, which maintains homeostasis of ABA levels but reduces ABA’s antitranspirant effect after exogenous application (Han et al., 2017). In contrast, ABA-receptor agonists usually show longer persistence than ABA after exogenous application (Cao et al., 2017; Vaidya et al., 2019). Numerous ABA-receptor agonists have been described during the last decade (Lozano-Juste et al., 2020; Vaidya and Cutler, 2022). Specifically, we have explored the derivative of quinabactin (QB)/ABA mimic 1 (AM1) that contains four fluorine atoms in the methylbenzyl ring and is named AMF4 for AM1 fluorine derivative 4 (Cao et al., 2017). AMF4 is a potent agonist of *Arabidopsis thaliana* (arabidopsis) PYR1, PYL1-PYL3, PYL5 and PYL7, and the structure of the ternary PYL2-AMF4-HAB1 complex reveals a better occupancy of the PYL2 ligand-binding pocket by AMF4 than AM1 (Cao et al., 2017). AMF4 was more effective than ABA in promoting the interaction of PYR1, PYL1, PYL2 and PYL7 with the PP2C HAB1 (Cao et al., 2017). AMF4 was also a ligand for two orthologs of PYL1 and PYL2 in soybean (*Glycine max*), which suggests that PYL receptors in crops can trigger ABA response after AMF4 application (Cao et al., 2017). The use of ABA-receptor agonists in agriculture is very promising, but detailed physiological analyses are still scarce in crops. In this work, we have explored the use of the ABA-receptor agonist AMF4 in tomato plants that were grown under water-limiting conditions (50% field capacity) and we have analyzed the protection conferred by the agonist treatment.

## Materials and methods

### Plant material and experimental conditions

*Solanum lycopersicum* L. (cv Robin) seedlings were obtained from a local vendor. Sowing was done in pots with an automatic seeder to ensure uniform germination and growth up to the stage of two true leaves (BBCH scale 12) in a greenhouse. Only seedlings of the same size that were well-rooted and disease-free were used for the experiments. After reaching the desired size, plant trays were placed in a growth chamber with controlled conditions: temperature 24°C ± 2, photoperiod 16-8h (light/dark), humidity 60-75%, and irradiance 300 µmols m^-2^ s^-1^.

### Seed germination assay

*Solanum lycopersicum* L. seeds were sterilized using 50% commercial bleach plus 0.02% Tween-20 for 1 hour followed by 5x5 minutes washes with sterile water. Seeds were sown on 150 mm petri plates with 4 layers of sterile filter paper previously wetted with 15 mL of control (0.1% DMSO), ABA, or AMF4 solution. 30 seeds per plate were sown in duplicate for each treatment (n=60). The plates were placed in a growth chamber under long day conditions and 23°C, and radicle emergence was scored on day 8.

### Plant growth under water deficit and agonist treatment

**Figure 1:**
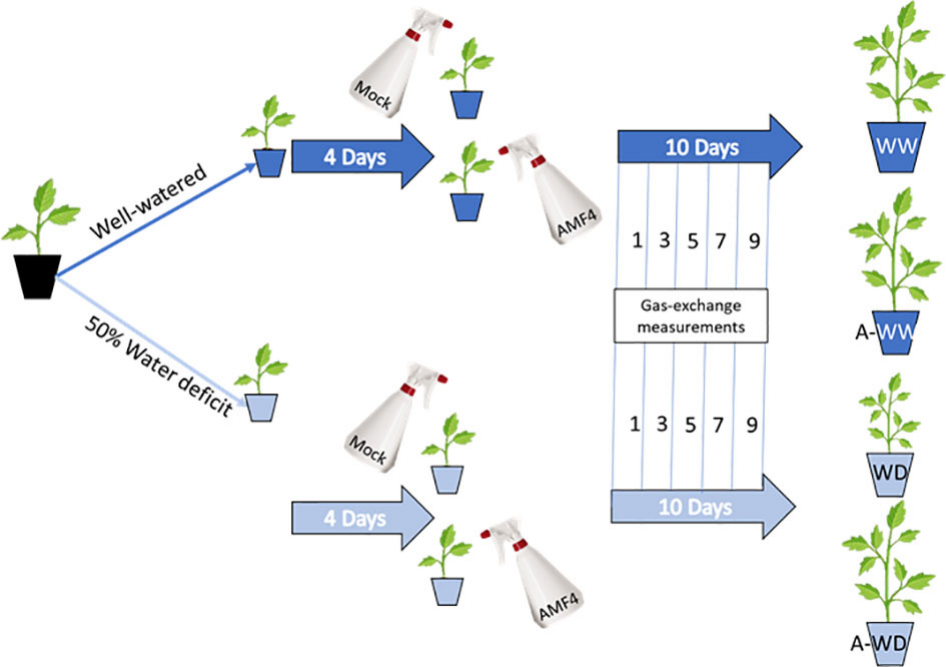
Treatments and experimental set up. Well-watered plants were mock- (WW) or 50 µM AMF4-treated (A-WW); plants subjected to water deficit were mock- (WD) or 50 µM AMF4-treated (A-WD). When a second application of AMF4 was performed (AA-WW or AA-WD), the plants received a second foliar spray at 6 days after the first one.

Plants with two true leaves were subjected to a growth experiment under well-watered (WW) or water deficit (WD) conditions, essentially following the procedure described by Jiménez-Arias et al. (2022b). Thus, WW plants received 10 ml/pot (full field capacity) whereas WD plants received 5 ml/pot each day for 14 days. Symptoms of WD were evident after 5 days and photosynthesis showed a dramatic decline after 11 days. Briefly, four groups of plants were established to test the effect of AMF4- versus mock-treatment, either under well-watered or water deficit conditions, and each experimental condition was tested in twenty plants (**Figure 1**). Plants designated as well-watered were irrigated to full field capacity, while those grown under water deficit conditions were irrigated with 50% less water. To ensure good nutrition, in all cases, plants were irrigated with a half-strength Hoagland solution (Hoagland and Arnon, 1950). Foliar agonist- or mock-treatments were carried out in 10 mM MES at pH 5.7 with 0.1% DMSO and 0.05% Tween 20, and additionally, 50 µM AMF4 was applied in agonist-treated plants. The treatment consisted of spraying 2.5 ml directly onto the tomato leaves to ensure that the entire leaf was soaked. The mock- or 50 µM AMF4-treatments were applied four days after water deficit imposition (**Figure 1**). Measurements of gas-exchange parameters were performed at 1, 3, 5, 7 and 9 days after foliar agonist- or mock-treatment.

### Growth measurements and stress index calculation

Two independent experiments were conducted with 23 plants for each treatment, and the plants were finally collected 14 days after the experiment start. Plants were dried in an oven at 60 °C for two days and the shoots and roots were weighed separately. Different indices were calculated using the weight of the plants at 14 days, such as the stress susceptibility index (SSI) (Ganança et al., 2015), stress tolerance index (TSI) (Farshadfar et al., 2013), relative growth rate (RGR), plant water use efficiency (WUEp), and relative water content (RWC) (Jiménez-Arias et al., 2022a; Jiménez-Arias et al., 2022b).

### Gas exchange measurement

Gas exchange analyses were carried out on the fully developed leaves (N = 30). Photosynthesis (Pn), intracellular CO_2_ (Ci), stomatal conductance (gs) and transpiration rate (E) were measured on the attached leaves using a portable infrared gas analyzer (LCPro, BioScientific Ltd., Hoddesdon, UK). The values for instantaneous water use efficiency (iWUE) and intrinsic water use efficiency (intWUE) are the ratios between Pn/E and Pn/gs, respectively (Seibt et al., 2008). The ratio between Pn and Ci was also calculated. The measurements were carried at ambient CO_2_ concentration, photosynthetic photon flux density (PPFD) of 1000 μmol m-2 s-1 (optimized with a light curve) and cuvette airflow of 500 mL min-1. Finally, water use efficiency at the total plant stage (WUEp) was measured as the ratio between biomass increment and the amount of water consumed (Medrano et al., 2015).

### Proline determination

The proline concentration at each experimental time-point was calculated as the average of 6 plants. Proline content was determined as described by Bates et al. (1973) with minor modifications (Jiménez-Arias et al., 2015). Samples of 20-50 mg of dry tissue (leaves) were ground and extracted with 4 ml of 3% sulphosalicylic acid. The plant extract was centrifuged at 15000 g for 30 minutes, and 2 μl of the supernatant was mixed with 2 ml of acid ninhydrin and incubated at 100°C for 60 minutes. This reaction was stopped in an ice bath. After extraction with 4 ml toluene, the absorbance of the organic phase was measured at 520 nm in an Aquarius CE7200 double-beam spectrophotometer (Cecil Instruments, Cambridge, England). The proline concentration was calculated from a standard curve and normalized to dry weight.

### PP2C activity assay

Phosphatase activity was measured using p-nitrophenyl phosphate (pNPP) as a substrate. The assays were performed in a 100 μL solution containing 25 mM Tris HCl [pH 8.0], 10 mM MnCl2, and 25 mM pNPP. The assays contained 1 μM phosphatase (ΔN-HAB1), 2 μM receptor and the indicated concentrations of ABA or AMF4. Phosphatase activity was recorded with a ViktorX5 reader at 405 nm every 60 s over 20 min, and the activity obtained after 20 min is indicated in the graphs. The His-tagged Sl08g076960, Sl06g061180 and Sl03g007310 ABA-receptor proteins were purified as described by González-Guzmán et al. (2014).

### Microscopic determinations of stomatal aperture

Six tomato plants were mock- or 50 μM AMF4-treated and epidermal peels were taken 24 hours after the treatment from abaxial and adaxial leaf surfaces and mounted in glycerin. Observations and photomicrographs were carried out using an Optika B-350 light microscope with a computer image capture system Moticam 2500. The stomatal aperture analysis was performed on the computer using Motic Images Plus 2.0 program, and 120 stomata from 6 different plants were measured for each treatment condition.

### Scanning electron microscopy

Six plants were mock- or 50 μM AMF4-treated and analyzed 24 hours after treatment. Two leaves were cut into small pieces (3 mm^2^), fixed in 2.5% glutaraldehyde in PB 0.05 M (pH 7), dehydrated in graded ethanol series, dried using 1,1,1,3,3,3-hexamethyldisilazane (HMDS) and coated with gold (15 nm). Observations and photomicrographs were made using a ZEISS EVO15 (ZEISS) scanning electron microscope (General Services for Supporting Research, University of La Laguna). Representative images observed in more than 90% of stomata were used in the figure.

### Analysis of macronutrients in tomato plants

Analyses of Macronutrients (Ca, K, Mg and P) were conducted using leaf samples from each treatment harvested at the end of the water deficit experiment. Samples were dried at 80 °C and then ground using an IKA M20 mill. The samples were then placed in an oven at 105 °C for five hours and then transferred to a desiccator to obtain the dry weight. Next, 500 mg of ground powder was taken from each tomato sample and after conversion to ash, the samples were treated with 6 N hydrochloric acid in a muffle furnace at 480 °C. Mineral content was determined using Avio^®^ 500 ICP-OES (Perkin Elmer) and interpolation of the data into a standard curve. N and S measurements were carried out on 1 mg dry leaf samples using the CHNS TruSpec Micro (LECO Corporation). All measurements were carried out in triplicate.

### Statistical analyses

One-way ANOVA tests (Duncan’s *post hoc*) were applied to analyze the differences between treatments in all measures studied. A two-way ANOVA was then run to test the differences among factors. Irrigation deficit was the first factor, AMF4 treatment the second, and interaction the third. All statistical studies were performed using IBM-SPSS24 statistical package.

### qRT-PCR

Ten-day-old tomato seedlings (cv. Moneymaker) were mock- or 10 μM AMF4-treated for 3 h. Total RNA was extracted using a NucleoSpin RNA plant kit. Synthesis of cDNA and quantitative real-time PCR (qRT-PCR) analyses were performed as described by González-Guzmán et al. (2014). Amplification of the ABA-responsive *Sl02g084850* (*SlRAB18*), *Sl06g067980* (*SlLEA*), and *Sl06g019170* (*SlP5CS1*) genes was done using the primers described by González-Guzmán et al. (2014). Expression was normalized using the values obtained with *Sl06g009970* (*SlEF1a*).

### AMF4 synthesis

AMF4 (N-(2-oxo-1-propyl-1,2,3,4-tetrahydroquinolin-6-yl)-1-(2,3,5,6-tetrafluoro-4-methylphenyl) methanesulfonamide) was synthesized from commercial 3,4-Dihydroquinolin-2(1*H*)-one following the protocols specified in literature (Cao et al., 2017).

## Results

### AMF4 activates PYL1-like receptors, inhibits seed germination, reduces stomatal aperture and upregulates ABA-responsive genes in tomato

**Figure 2:**
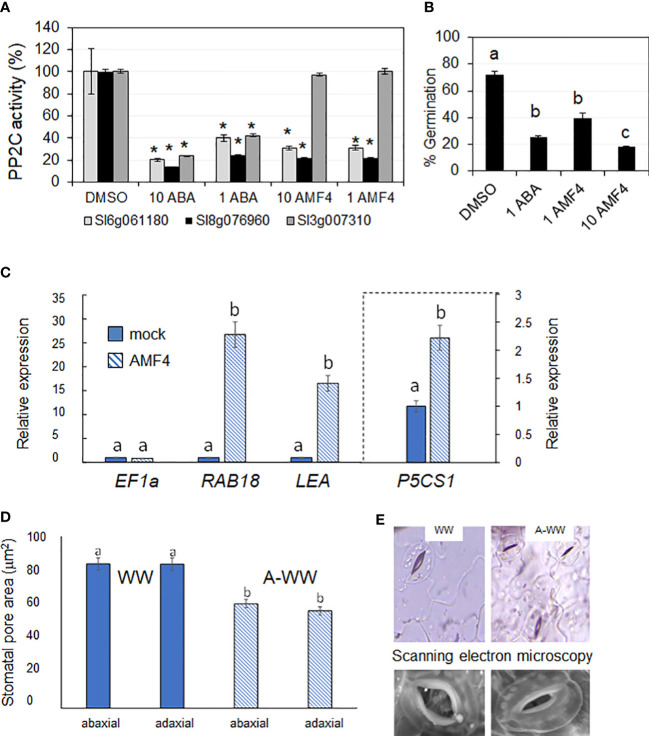
AMF4 activates PYL1-like ABA receptors, inhibits tomato seed germination, induces ABA responsive genes and stomatal closure. **(A)** PP2C inhibition assay in absence (0.1% DMSO) or in the presence of either 1 or 10 μM ABA or AMF4 and the indicated tomato ABA receptors. Values represent mean ± SD of 2 assays. Asterisks indicate statistical significance (p<0.05) in Student’s *t* test compared to its corresponding DMSO-treated receptor. **(B)** Inhibition of tomato seed germination by ABA or AMF4. Seeds were sown on ABA, AMF4 or control conditions (0.1% DMSO) and germination was scored 8 days later. Values represent mean ± SD of 2 assays. Different letters indicate statistical significance by One-test ANOVA (p<0.05). **(C)** AMF4 upregulates ABA-responsive genes in tomato. AMF4 treatment upregulates the expression of *SlRAB18*, *SlLEA* and *SlP5CS1*. Ten-day-old tomato seedlings were either mock- or 10 μM AMF4-treated for 3 h. The histograms indicate the relative induction by AMF4 treatment of the indicated tomato genes with respect to mock treatment (value 1). *SlEF1a* expression was used to normalize the expression of ABA-responsive genes. **(D)** AMF4 induces stomatal closure in tomato. The area of the stomatal pore was measured before and after AMF4 treatment in WW conditions as indicated in methods. Different letters indicate statistical significance by One-test ANOVA (p<0.05). Values represent mean ± SE. **(E)** Microscopy analyses show stomatal closure in response to AMF4 treatment (A-WW). Top panel, light microscope images from mock- and AMF4-treated tomato leaves at 24 h post-treatment. Bottom panel, scanning electron microscopy images from mock- or AMF4-treated tomato leaves at 24 h post-treatment.

In order to characterize the activity of the ABA-receptor agonist AMF4 in tomato, firstly, we tested the *in vitro* activity of AMF4 using two tomato ABA receptors that are orthologs of AtPYL1, namely Sl06g061180 and Sl08g076960. The cloning and purification of the corresponding recombinant proteins were described previously (González-Guzmán et al., 2014). As a result, we found that both PYL1-like tomato receptors were activated by AMF4, resulting in the inhibition of the PP2C HAB1 (**Figure 2A**). AMF4 does not activate PYL8, and indeed, the tomato ortholog of PYL8, namely Sl03g007310, did not inhibit the activity of the PP2C HAB1 even at 10 μM AMF4 (**Figure 2A**). These results confirm that AMF4 can activate certain tomato ABA receptors *in vitro*.

ABA plays an important role in the maintenance of seed dormancy and exogenous application of ABA leads to inhibition of germination (Cornforth et al., 1965). To test the *in vivo* activity of AMF4 compared to ABA, we performed seed germination assays on filter paper wetted with ABA or AMF4. ABA- or AMF4-treated seeds showed a similar percentage of germination inhibition at 1 µM (**Figure 2B**). Moreover, 10 µM AMF4 inhibited tomato seed germination by more than 80%, thus confirming AMF4’s activity in tomato (**Figure 2B**). AMF4 regulates ABA-responsive genes in arabidopsis plants and reduces transpiration both in arabidopsis and soybean plants as a result of AMF4-induced stomatal closure (Cao et al., 2017). In order to test the biological activity of AMF4 in tomato seedlings, 10-day-old plants were treated with 10 μM AMF4 for 3 h and subsequently, we analyzed transcript levels of three tomato genes by qRT-PCR, namely *RESPONSIVE TO ABA 18 (RAB18)*, *LATE EMBRYOGENESIS ABUNDANT (LEA) 6g067980* and the *Δ1-PYRROLINE-5-CARBOXYLATE SYNTHASE (P5CS1)* genes. These genes were upregulated 26, 16 and 2-fold by AMF4 treatment, respectively (**Figure 2C**). Given that P5CS1 is a rate-limiting enzyme for the accumulation of proline, its upregulation suggests that AMF4 treatment might have a positive effect to increase the levels of this metabolite (Bhaskara et al., 2015; see below). Osmotic adjustment and regulation of transpiration can improve plant adaptation in water-limited environments (Bhaskara et al., 2015). Therefore, we also measured the stomatal aperture of tomato leaves using whole-leaf imaging before and after 50 μM AMF4-treatment. AMF4 induced stomatal closure in tomato and the stomatal pore area of AMF4-treated leaves was 28% and 32% lower in the adaxial and abaxial sides of the leaf, respectively, compared to mock-treated plants (**Figure 2D**). Additionally, we obtained stomatal images by both light and scanning electron microscopy, which show a reduction in stomatal aperture after AMF4 treatment (top and bottom panels, respectively, **Figure 2E**).

### AMF4 enhances tomato tolerance against water deficit

**Figure 3:**
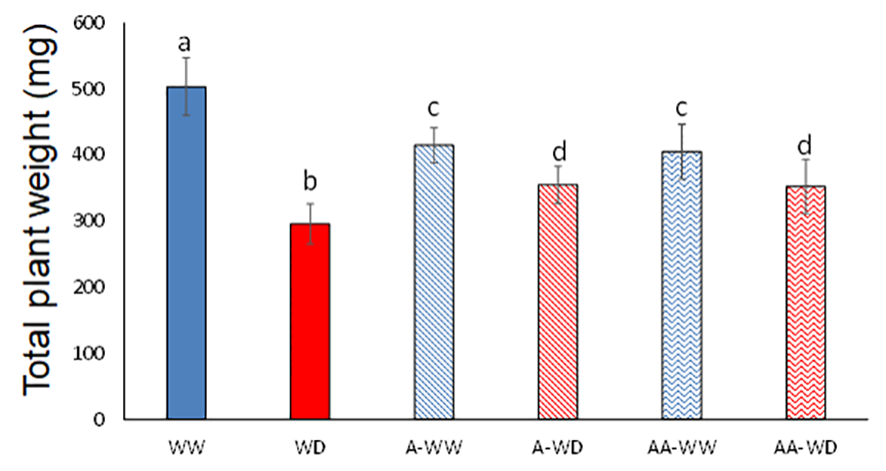
AMF4 enhances plant growth under WD conditions. Plants subjected to water deficit (50% field capacity) were mock- or 50 µM AMF4-treated at 4 days after setting up stress conditions. When a second application of AMF4 was performed (either AA-WW or AA-WD), the plants received a second foliar spray 6 days after the first one. Total plant weight at the end of the experiment (14 days) is shown. Different letters indicate statistical significance by One-test ANOVA (p<0.05).

In nature, water is possibly the most limiting factor for plant growth and development. Water deficit negatively affects photosynthesis (Luo et al., 2016) and plant nutrient uptake (Coelho et al., 2023), and overall reduces plant growth and final crop yield (Shao et al., 2008). In order to test the potential use of AMF4 to increase drought tolerance we subjected tomato plants to a water deficit regime for 14 days as described in methods and **Figure 1**. The negative impact on tomato growth after a 50% reduction in irrigation for two weeks was measured, resulting in a 42% reduction in plant growth at the end of the experiment (**Figure 3**; **Supplementary Table 1**). In WW plants, treatment with 50 μM AMF4 had a negative effect on plant growth and resulted in an 18% growth reduction compared to mock-treated WW plants (**Figure 3**). The growth-repressing effect of ABA signaling is well known and it represents a trade-off for stress adaptation (Miao et al., 2018). Thus, in the absence of a water deficit, the use of AMF4 needs further evaluation. For example, proper use of AMF4 might lead to a reduction of vegetative growth but with minimum effect on the yield of some crops, thus increasing the harvest index. In contrast, it is noteworthy that AMF4-treated plants that were exposed to water deficit showed a significant 17% increase in plant weight compared to mock-treated (**Figure 3**). We also tested the effect of a second AMF4 treatment (AA-WW or AA-WD), applied 6 days after the first one, but the results showed no significant difference with the plants treated only once (**Figure 3**). Therefore, AMF4 application under water deficit conditions improves plant growth of tomato plants compared to mock-treated plants (**Figure 3**).

In addition to a direct measurement of plant weight to evaluate plant tolerance to water deficit, we have used the stress susceptibility index (SSI) (Fischer and Maurer, 1978), stress tolerance index (STI) (Rosielle and Hamblin, 1981) and relative growth rate (RGR) (Hunt et al., 2002). SSI was proposed by Fischer and Maurer (1978) to measure yield stability, which includes changes in potential and actual yields in variable environments. The stress tolerance index (STI) was defined by Rosielle and Hamblin (1981) as the differences in yield between plants grown under water deficit or well-watered conditions. Finally, RGR shows how the plant increases weight in a certain period (Hunt et al., 2002). The increase in plant tolerance to water deficit by AMF4 treatment was evident when we used the mentioned indices (**Table 1**). Thus, according to the RGR, A-WW plants have a daily growth reduction of 1% compared to the mock-treated WW plants, while A-WD plants show the opposite behavior compared to mock-treated in WD conditions. WUEp has been defined as the ratio between plant biomass produced and the water used in a period of time (Stanhill, 1986). In WD conditions, A-WD plants showed a 25% increase in WUEp compared to mock-treated, indicating that AMF4 improves biomass production per unit of water consumed by tomato seedlings (**Table 1**). Concerning AMF4 treatment in the absence of water deficit, although RGR was reduced, proper use might benefit agronomic yield as indicated above. An important indicator of plant water status is the RWC (Soltys-Kalina et al., 2016). A comparison of RWC values in WD and A-WD plants indicated that AMF4 treatment led to improved water status of the plant under water deficit (**Table 1**). Moreover, A-WD plants reached a similar RWC as WW plants, whereas mock-treated WD plants showed a significant 20% lower RWC value compared to WW plants. In agreement, A-WD plants showed a lower SSI index compared to WD plants, indicating higher stress tolerance, because lower values of the SSI index are positively correlated with higher yields under stress (Farshadfar et al., 2013; Zdravkovic et al., 2013). The higher values of the STI index in A-WD plants indicated that AMF4 significantly increased tolerance to water deficit stress. Finally, the effect of WD and AMF4 treatment was tested either individually or considering their mutual interaction through a two-way ANOVA analysis, in which we compared the dry weight of whole plants, shoots or roots (**Supplementary Table 1**). The WD had a significant effect on the weight of whole plants, shoots or roots, but the AMF4 treatment did not produce a significant effect on the rootweight, compared to WW conditions, suggesting that the agonist had the greatest influence on shoot growth. Interestingly, the interaction showed differences between A-WD in all variables studied, and the F-values and statistical significance showed that the interaction is higher in shoots, showing that the treatment has a direct and positive interaction in plant acclimation under water deficit stress (**Supplementary Table 1**).

**Table 1:** Growth and stress indices.

	RGR	WUE_p_	RWC_14D_	SSI	STI
WW	0.11	2.8	89.1 ± 8.7 a		
A-WW	0.10	2.2	90.1 ± 6.8 a		
WD	0.08	2.8	71.4 ± 8.8 b	2.5	0.5
A-WD	0.09	3.7	85.5 ± 8.1 a	1.0	0.81

Values labeled by different letters indicate significant differences at p<0.05. The abbreviations are defined in the text and the relative water content was calculated at the end of the experiment (RWC_14D_).

### AMF4 treatment improves CO_2_ assimilation and water use efficiency under WD conditions

**Figure 4:**
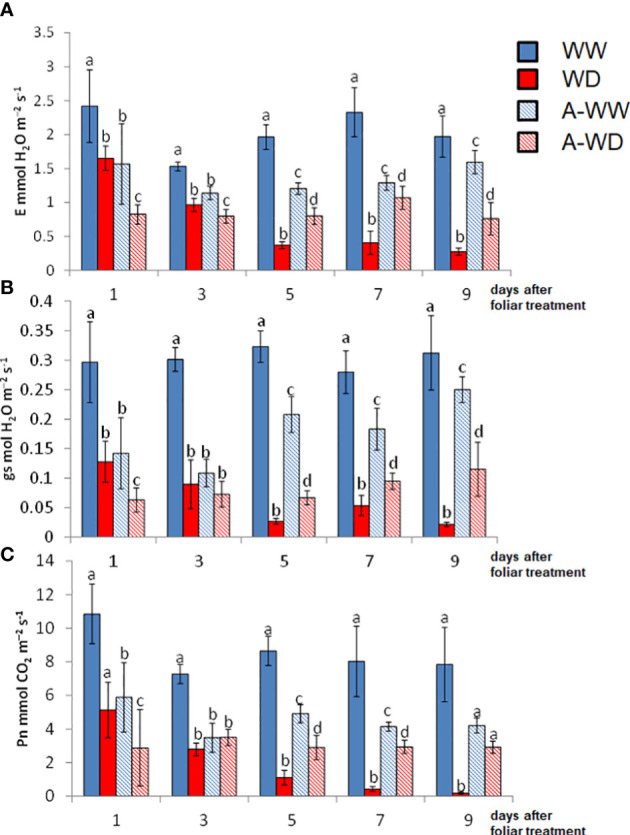
Plant gas exchange measurements and CO_2_ assimilation along the experiment. **(A)** Transpiration (E). **(B)** Stomatal conductance (gs). **(C)** Net photosynthesis (Pn). Bars labeled by different letters indicate significant differences at p<0.05.

ABA treatment has direct effects on leaf gas-exchange parameters by promoting stomatal closure, which directly affects water balance (Li and Liu, 2021). Plants benefit from ABA-induced stomatal closure to reduce transpiration during water deficit stress (Lee and Luan, 2012). As expected for an ABA-receptor agonist that mimics the ABA phytohormone, AMF4 had a clear effect on gas-exchange parameters, as shown by the E, gs and Pn values (**Figure 4**). Firstly, AMF4 treatment in WW plants led to a reduction of gs that was persistent throughout the experiment (**Figure 4B**). Initially, this reduction was similar to that imposed by WD, but 5 days after foliar treatment, the reduction of gs and E by WD was higher than that achieved by AMF4 treatment in WW plants (**Figures 4A, B**). Interestingly, the dramatic decrease in photosynthesis induced by WD conditions was partially mitigated in AMF4-treated plants subjected to WD, see for example Pn values at 5, 7 and 9 days (**Figure 4C**). These results can explain the higher growth under WD conditions of AMF4-treated plants compared to mock-treated plants (**Figure 3**), as well as the higher RWC and the improved stress tolerance indexes under WD conditions (**Table 1**).

**Figure 5:**
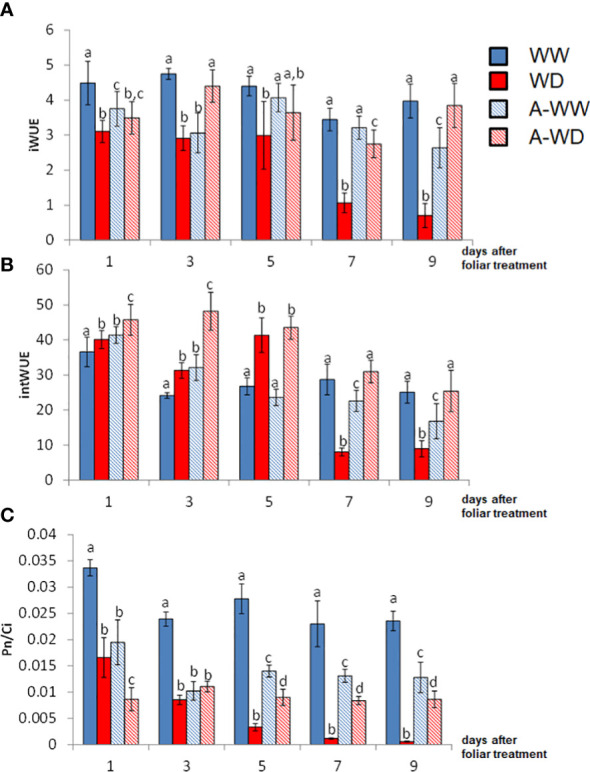
Enhanced WUE and CO_2_ assimilation ratio by AMF4 treatment under WD conditions. **(A)** Instantaneous water use efficiency, Pn/E. **(B)** Intrinsic water use efficiency, Pn/gs. **(C)** Pn/Ci index. Bars labeled by the same letter did not show significant differences at p<0.05. Ratios and standard deviations were obtained from values reported in **Figure 4**.

Tomato plants under WD conditions showed a dramatic decrease in gs and E, as well as in Pn. The activation of ABA signaling by AMF4 was able to mitigate the drop in Pn and led to a reduction of gs, which increased iWUE and intWUE values (**Figures 5A, B**). These parameters are crucial to maintaining crop yield under WD field conditions (Condon, 2004). Thus, AMF4-treated plants under WD conditions showed higher water use efficiency compared to mock-treated WD plants as shown by intWUE and iWUE values (**Figures 5A, B**). These ratios are not always directly correlated with WUEp (Medrano et al., 2015; Yang et al., 2019); however, in our WD conditions, AMF4-treated plants also showed higher WUEp compared to mock-treated WD plants (**Table 1**). It is interesting to point out that the A-WW plants had a lower intWUE value compared to the WW plants. This ratio, which reflects CO_2_ assimilation versus water loss, shows again that the AMF4 treatment negatively affects CO_2_ assimilation when the plant is not under stress conditions. The Pn/Ci ratio also provides a simple method for assessing the efficiency of CO_2_ assimilation according to the intracellular CO_2_ concentration (Long, 2003). The AMF4 treatment led to plant protection in plants exposed to WD, as shown by the higher value of Pn/Ci compared to mock-treated WD plants (**Figure 5C**). In summary, AMF4 treatment improved water stress avoidance, therefore tomato plants showed better water balance and CO_2_ assimilation; therefore, might be a promising approach to maintain yield under drought conditions.

### AMF4 treatment enhances proline accumulation and macronutrient content

**Figure 6:**
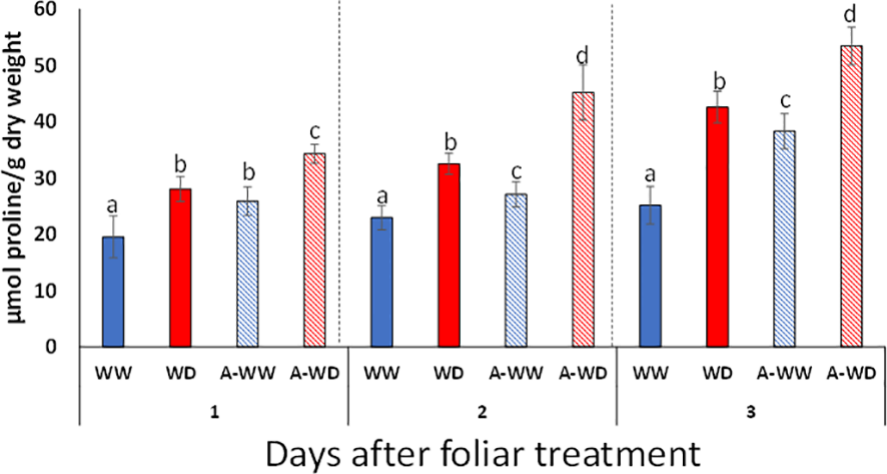
Enhanced proline accumulation induced by AMF4 treatment under WD conditions. Bars labeled by different letters indicate significant differences at p<0.05.

Osmotic adjustment is a plant adaptive response to water deficit and requires the intracellular accumulation of compatible solutes to facilitate water uptake, protect the integrity of cellular structures and prevent the denaturation of soluble enzymes (Alvarez et al., 2022). Proline is accumulated in a wide range of taxonomically diverse plants, including tomato (Alvarez et al., 2022). Either ABA or ABA-receptor agonists such as quinabactin are able to upregulate the expression of the *P5CS1* gene, which encodes a rate-limiting enzyme for proline biosynthesis (Szekely et al., 2008; González-Guzmán et al., 2014). AMF4 also upregulated *P5CS1* expression in tomato (**Figure 2C**). In order to evaluate the effect of AMF4 treatment on proline levels, we measured proline at 24, 48 and 72 h in WW, A-WW, WD and A-WD plants (**Figure 6**). The combination of water deficit and AMF4 treatment enhanced proline levels compared to mock-treated plants subjected only to WD (**Figure 6**). The additional boosting of proline biosynthesis might reflect an additive effect of both osmotic stress and AMF4 on *P5CS1* upregulation.

Water deficit stress leads to impairment of nutrient uptake, also affecting transport and distribution, which finally downregulates mineral nutrition and impairs nutrient partitioning (Sánchez-Rodríguez et al., 2010; D’Oria et al., 2022). We analyzed the nutrient content of Ca, K, Mg P, N and S, both in shoots and roots of the four groups established in our experimental conditions. WD significantly reduced the content of Ca, K and Mg in shoots, and uniquely of K in roots (**Table 2**). AMF4 treatment significantly prevented this reduction in nutrient content in both tissues. Thus, the treatment maintains potassium levels, which are crucial for osmotic adjustment. Proper K levels are also required for stomatal regulation and to maintain dry matter accumulation under drought stress (Wang et al., 2013). On the other hand, adequate Ca levels are required for different processes involved in plant response to water stress, such as plasma membrane function, cell wall structure and signaling (Tonetto de Freitas et al., 2014). Specifically, in tomato Ca deficiency leads to disorders in fruit such as blossom-end rot (BER), and foliar application of ABA prevented BER disorder (Tonetto de Freitas et al., 2014). Spraying with ABA increased apoplastic Ca concentrations and reduced BER incidence (Tonetto de Freitas et al., 2014). Interestingly, AMF4 treatment recovered Ca levels in shoots of plants that were subjected to water deficit (**Table 2**). Finally, it is interesting to note the significant increase observed in nitrogen concentration in shoots after the application of the agonist under WW conditions, while the comparison of WD and A-WD plants did not reveal a significant difference (**Table 2**).

**Table 2:** Tomato shoot and root macronutrient analyses.

Tissue	Treatment	% (weight/weight)					
		Ca	K	Mg	P	N	S
**Shoots**	**WW**	1.3± 0.02 a	1.4± 0.01 a	0.37± 0.02 a	0.14± 0.01 a	1.1± 0.05a	0.3± 0.04 a
	**A-WW**	1.4± 0.01 b	1.8± 0.02 b	0.39± 0.02 b	0.15± 0.01 a	1.5± 0.04b	0.4 ± 0.05 a
	**WD**	1.1± 0.01 c	1.2± 0.01 c	0.35± 0.01 c	0.14± 0.01 a	1.3± 0.1 a, b	0.4 ± 0.02 a
	**A-WD**	1.3± 0.01 a	1.4± 0.02 a	0.37± 0.01 a	0.15± 0.01 a	1.5± 0.05 b	0.5 ± 0.05 a
**Roots**	**WW**	0.7± 0.02 a	1.4± 0.02 a	0.6± 0.02 a	0.16± 0.01 a	1.5± 0.1 a	0.3± 0.02 a
	**A-WW**	0.5± 0.01 b	1.5± 0.01b	0.5± 0.02 b	0.18± 0.01 a	1.6± 0.03 a	0.2± 0.1 a
	**WD**	0.7± 0.01 a	1.3± 0.01 b	0.6± 0.01 a	0.15± 0.01 a	1.4± 0.1 a	0.3± 0.05 a
	**A-WD**	0.8± 0.02 c	1.5± 0.02 b	0.6± 0.01 a	0.18± 0.01 a	1.6± 0.08 a	0.4± 0.04 a

Values labeled by different letters indicate significant differences at p<0.05. The percentage (%) indicates the total amount of the macronutrient (g) per 100 g plant dry tissue.

## Discussion

ABA-receptor agonists are promising to activate the plant adaptive response against abiotic stress and overcome the shortcomings of exogenous ABA application, i.e. rapid catabolism and light-induced isomerization that inactivate ABA (Gao et al., 2016; Han et al., 2017). The development of agonist molecules that mimic ABA action has witnessed a marked advance during the last decade; however, still few reports have documented their use in crops, including detailed physiological analyses (Vaidya et al., 2019; Hewage et al., 2020). In this work, we have explored the protection conferred by AMF4 in tomato during vegetative growth, paying attention to the agonist effect on the regulation of transpiration and assimilation of CO_2_ under conditions of water deficit. Previously, AMF4 was reported to induce cold tolerance in wheat and reduce water loss in *Spinacia oleracea* (spinach), which contributed to maintaining visual quality and extending the shelf-life of this leafy vegetal (Ma et al., 2018; Li et al., 2020). Thus, in addition to protecting crops from abiotic stress, the use of an ABA-receptor agonist can play a protective role at the postharvest stage.

AMF4 was able to protect arabidopsis and soybean plants after water withdrawal, increasing plant survival (Cao et al., 2017). Instead of such severe drought treatment, we have applied here a water deficit regimen that impairs tomato growth but does not compromise the survival of the plant. Thus, we aimed to investigate protection conferred by AMF4 in water deficit conditions that impair photosynthesis and reduce biomass production but are not so severe as a complete water withdrawal (Cao et al., 2017). First of all, we found that AMF4 binds and activates at least two PYL1-like tomato receptors; therefore, we confirmed that crop ABA receptors can be activated by this agonist. AMF4 showed *in vivo* effect to inhibit seed germination, induced stomatal closure in tomato leaves and upregulated the expression of ABA-responsive genes (**Figure 2C**). Then, when a water deficit regimen (50% reduction versus well-watered plants) was applied for 14 days, we found that AMF4 treatment improved plant growth and increased the RWC of the agonist-treated compared to mock-treated plants. To investigate the physiological response of tomato plants during this partial drought period, we measured plant gas exchange as indicated in **Figure 4**. As expected from the agonist’s capability to induce stomatal closure, 1 day after application, AMF4-treated plants showed reduced gs and Pn compared to mock-treated plants; however, subsequently the AMF4 treatment was efficient to maintain transpiration and photosynthesis. Thus, whereas mock-treated plants experienced a sharp decline in transpiration and photosynthesis, AMF4-treated WD plants showed higher parameters of transpiration and photosynthesis at 5, 7 or 9 days after application (**Figure 4**). On the contrary, when plants were subjected to water deficit and not protected by the agonist, photosynthesis fell dramatically. As a result, the parameters of WUE (both iWUE and intWUE) were markedly improved in tomato plants subjected to water deficit and treated with AMF4 (**Figure 5**). These results indicate that the intrinsic tradeoff between water lost by transpiration and assimilation of CO_2_ is well regulated by the agonist treatment. Moreover, in the absence of agonist treatment, sustained water deficit along the experiment resulted in a sharp decline in photosynthesis and lower WUE. Under our experimental conditions (50% water deficit for 14 days), mock-treated plants suffered a sharp decline in WUE parameters because Pn dropped dramatically and the concomitant reduction in E was not enough to increase WUE. It is possible that in a different experimental set for testing the drought effect in tomato, in which photosynthesis was less impaired, the concomitant reduction in E could lead to an increase of WUE parameters. This was not recorded under our experimental conditions because measurements of gas-exchange parameters were performed after a sustained water deficit.

The agonist showed a long-lasting effect in the aforementioned physiological analyses, which suggests persistent activation of ABA signaling. AMF4 activates ABA signaling in tomato through at least two PYL1-like ABA receptors (**Figure 2A**), which are expected to inhibit clade A PP2Cs upon perception of the ligand. In *Arabidopsis thaliana*, mutants impaired in Highly ABA-induced (HAI) phosphatases, which belong to clade A PP2Cs, showed enhanced proline content under osmotic stress conditions (Bhaskara et al., 2012). HAIs and other clade A PP2Cs are conserved in tomato and might be inactivated by AMF4 treatment (Sun et al., 2011; González-Guzmán et al., 2014). Thus, we wondered whether another critical response to cope with water deficit, i.e. osmotic adjustment through the accumulation of compatible solutes such as proline, was affected by the agonist treatment. To this end, we measured proline accumulation (**Figure 6**), because this compound is a key marker for the adaptive plant response against abiotic stress and its accumulation is induced by ABA, osmotic or salt stress (Szekely et al., 2008; Bhaskara et al., 2015). Additionally, proline also plays a role to detoxify reactive oxygen species and for the protection of protein structures through chaperon-like properties (Hong et al., 2000; Signorelli et al., 2014). As expected, we found that proline concentration increased after the water deficit compared to measurements in well-watered plants (**Figure 6**). Interestingly, AMF4 treatment enhanced proline levels over those achieved in mock-treated plants subjected to water deficit; therefore, an additional increase in proline levels is obtained by the agonist effect. Drought stress induces proline accumulation through the upregulation of pyrroline-5-carboxylate synthetase (P5CS), specifically, the P5CS1 isoform is stress-inducible (Szekely et al., 2008). Given that *P5CS1* upregulation induced by drought stress occurs through both ABA-dependent and ABA-independent pathways, we assume that water deficit and AMF4 cooperate to induce *P5CS1* through both pathways and therefore higher proline levels are produced (Savouré et al., 1997; Strizhov et al., 1997; Alvarez et al., 2022).

## Conclusions

**Figure 7:**
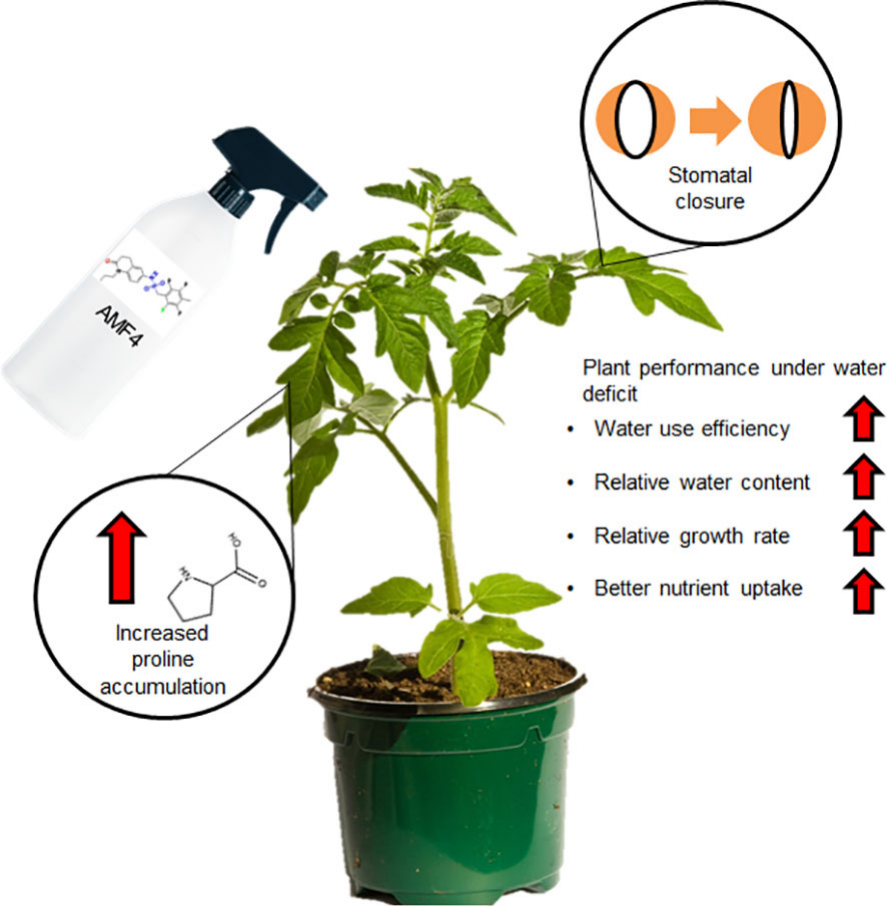
Summary of the AMF4 effects in tomato and the enhanced plant performance after agonist treatment under water deficit stress.

This study implies that molecules developed in the model plant *Arabidopsis thaliana* as ABA-receptor agonists can be used in crops such as tomato to improve plant growth under water deficit. Thus, we describe in this work a protective effect of AMF4 on the vegetative growth of tomato, which correlates with enhanced CO_2_ assimilation, higher levels of proline accumulation and improved content of certain macronutrients. The molecular mechanism of AMF4 action involves, at least, the regulation of ABA-responsive genes and induction of stomatal closure. We have summarized the major effects observed after the agonist treatment in **Figure 7**, which contribute to the improved plant performance under water deficit reflected in increased WUE, RWC, RGR and drought avoidance through regulation of stomatal closure. Future experiments should address the effect of this agonist treatment on fruit yield and quality under water deficit conditions to further validate this approach for tomato production by farmers.

## Data availability statement

The original contributions presented in the study are included in the article/**Supplementary Material**. Further inquiries can be directed to the corresponding authors.

## Author contributions

Conceptualization, DJ-A and PR. Methodology, DJ-A, SM-S, ES, JL-J, AC, JE, AB and PR. Investigation, DJ-A, SM-S, ES, JL-J, AC, JE, AB and PR. Visualization, DJ-A, SM-S, ES, JL-J, AC and PR. Writing original draft preparation, DJ-A and PR. Writing—review and editing, input from all authors; Supervision, DJ-A, AB and PR. Funding acquisition, AB. and PR. All authors contributed to the article and approved the submitted version.
