# Supplementary material for: Abscisic acid mimic-fluorine derivative 4 alleviates water deficit stress by regulating ABA-responsive genes, proline accumulation, CO2 assimilation, water use efficiency and better nutrient uptake in tomato plants
Source: Front Plant Sci. 2023 Jun 8;14:1191967. doi: 10.3389/fpls.2023.1191967 (PMC10285300; doi:10.3389/fpls.2023.1191967)
Supplement: Supplementary file 1 [file Table_1.docx]

Supplementary Material

Abscisic acid mimic-fluorine derivative 4 alleviates water deficit stress by regulating ABA-responsive genes, proline accumulation, CO2 assimilation, water use efficiency and better nutrient uptake in tomato plants

**David Jiménez-Arias*, Sarai Morales-Sierra, E. Suárez, Jorge Lozano-Juste, Alberto Coego, Juan Carlos Estevez, Andrés A. Borges, Pedro L. Rodriguez***

*** Correspondence:** [prodriguez@ibmcp.upv.es](mailto:prodriguez@ibmcp.upv.es); [david.j.a1983@gmail.com](mailto:david.j.a1983@gmail.com)

**Supplementary Table 1.**Results of a two-way analysis of variance for tomato plants submitted to different treatments as indicated in Figure 1, shoot and root dry weight, the factors ‘deficit irrigation’ and ‘agonist treatment’ and their interaction.

|  |  | | | | **Treatment** | **Variable** | | |
| --- | --- | --- | --- | --- | --- | --- | --- | --- |
|  |  | | |  |  |  | | |
|  |  |  |  |  |  | **Root weight** | **Shoot weight** | **Plant weight** |
|  | |  |  | **WW** | | 98.8 ± 20 | 404.7 ± 66 | 503.2 ± 80 |
|  | |  |  | **WD** | | 72.9 ± 18 | 222.7 ± 42 | 295.7 ± 49 |
|  | |  |  | **A-WW** | | 87.9 ± 11 | 326.5 ± 41 | 414.5 ± 89 |
|  | |  |  | **A-WD** | | 82.8 ± 36 | 272 ± 36 | 354.2 ± 78 |
|  |  |  |  | Two-way anova analyses | | | | |
|  |  |  |  | Dependent variable | | WD | A | WD + A |
|  |  |  |  | Plant dry weight | | 182.7 *** | 33.8*** | 53.1*** |
|  |  |  |  | Shoot dry weight | | 182.7 *** | 39.2*** | 9.8*** |
|  |  |  |  | Roots dry weight | | 167.8*** | 1.7 n.s. | 51.2*** |

Numbers represent F values: ***p < 0.001; n.s., non-significant
